# Factors impacting participation in research during the COVID-19 pandemic: results from a survey of patients in the ophthalmology outpatient department

**DOI:** 10.1186/s13063-022-06748-1

**Published:** 2022-09-30

**Authors:** Dalia Abdulhussein, Timothy E. Yap, Haider Manzar, Serge Miodragovic, Francesca Cordeiro

**Affiliations:** 1 Imperial College Ophthalmic Research Group (ICORG), Imperial College London, Exhibition Road, London, NW1 5QH UK; 2 Imperial College London, Exhibition Road, London, SW7 2AZ UK; 3 Glaucoma & Retinal Neurodegeneration Research Group, Institute of Ophthalmology, University College London, London, EC1V 9EL UK

**Keywords:** Trial recruitment, Patient and Public Involvement, COVID-19, Patient attitudes to trials

## Abstract

**Background:**

Understanding public and patient attitudes to clinical research is paramount to successful recruitment. The COVID-19 pandemic has led to additional hurdles in achieving this. Our aim is to understand the current factors and attitudes towards clinical trial participation in order to assist in recruitment to clinical trials.

**Methods:**

We conducted face-to-face interviews with patients in the outpatient department at a tertiary eye hospital facilitated by a 32-item questionnaire developed by the research team. Patient characteristics were correlated with their responses, in addition to qualitative thematic text analysis.

**Results:**

A total of 53 patients were interviewed. Forty per cent indicated that they would be willing to participate in clinical research in the current climate. General motivating factors for involvement in research included personal gain, altruism and contribution to innovation. Factors limiting participation included concerns regarding own safety, inconvenience, accessibility and lack of benefit. 22.6% of participants felt that the COVID-19 pandemic has changed their outlook on research. These were categorised into positive (increased awareness of the importance and need for research, altruism) and negative (increased anxiety, need to minimise exposure to the hospital environment) influences.

**Conclusions:**

Factors influencing patients’ decisions to participate in trials are similar to those observed prior to COVID-19 but with an increased focus on the environment the research is conducted in. The COVID-19 pandemic has had positive and negative impacts on patient attitudes towards research. Trial design, with a particular focus on setting and safety measures, in reassuring patients is increasingly important.

**Supplementary Information:**

The online version contains supplementary material available at 10.1186/s13063-022-06748-1.

## Key messages

- Recruitment to clinical trials is challenging under normal circumstances, and understanding patient and public attitudes to clinical research is critical for trial design.
- Previous studies have found that motivating factors for trial participation were mainly personal gain and altruism whilst deterrent factors were inconvenience and time commitment.
- We found that COVID-19 had both negative and positive impacts on patient attitudes to trial participation.
- Trial design and ensuring patient safety and minimising the risks of exposure to COVID-19, and highlighting these implementations are key for researchers wishing to resume trials in the post-COVID-19 era.

## Introduction

Clinical trials are paramount to medical research, with randomised control trials (RCTs) sitting at the top of the hierarchy of evidence pyramid [1]. The power of a study is the probability that it will reject a false null hypothesis, and therefore, as the power increases, the chance of type II error decreases. In order to yield a statistically significant result, a large sample size must be recruited [2–4]. It is also important that the sample size is representative of the target population. Therefore, it is important to optimise recruitment to clinical trials, and it can be said that factors influencing clinical research are ultimately dependent upon patient willingness to participate and commit to a study, and thus gaining a better understanding of patient views on trial participation is integral to trial recruitment.

Previous studies have demonstrated several factors that can influence patient willingness to participate in research such as cost, convenience, risks and benefits, nature of the trial and motivation [5]. A systematic review of three biological databases over a 10-year span found 78 papers reporting clinician and patient barriers. Of these, the most common patient barriers were time demands of the trial, patient preferences, worry due to uncertainty and lack of information or consent [6]. By overcoming these barriers, we can indirectly improve the efficacy of results by reducing factors such as attrition bias. Patient-centred approach to trials in the form of Patient and Public Involvement (PPI) can enhance the focus of clinical trials on the needs of patients, improve recruitment and thereby raise the quality of findings and helps their dissemination [5].

Furthermore, by involving patients in the design and conduct of trials, it ensures that clinicians and participants share similar expectations, avoiding misunderstandings that can negatively impact the results. If researchers and patients differ in motives and expectations, it can reduce participation [7]. A recent study on patient motivation for study participation found that those most willing to be recruited were incentivised by a genuine interest to improve treatment, to help others and to support PPI and financial gain [7]. The most discouraging factors for patients focused on their expectations, with a lack of clarity on their roles and the study [7]. In an era where patient-centred care has come to the forefront of clinical practice, it may yield great benefit to incorporate similar practices in research.

The current coronavirus 19 (COVID-19) pandemic has had significant impacts on research and recruitment to trials [8]. In this study, we aim to ascertain which factors influence the patient’s decision to participate in clinical trials as well as to understand what the effect of the COVID-19 pandemic is on these perceptions, particularly in the setting of ophthalmology.

## Methods

### Questionnaire development

A 32-item questionnaire (Supplementary file 1) was developed based on a literature review and the author’s experiences of clinical trial recruitment [5, 9–18]. Twelve items were related to demographic and personal information. Eleven items were related to patient involvement in clinical trials and factors contributing to participation (or lack of). Eight Likert scale items were used to ascertain patient views on participation in clinical trials. Questions consisted of a mix of multiple-choice, free-text and 5-point Likert scale responses with the opportunity for further comments at the end.

The study was submitted as a service evaluation to our local institution for approval and was approved by the Imperial College Health Trust committee for clinical research. Since this was a service evaluation to evaluate patients’ perspectives of partaking in ophthalmological research during the height of the COVID-19 pandemic, ethical review by an external review board was, therefore, not required.

### Setting and study participants

The questionnaire was developed for use in ophthalmology patients. Therefore, patients were approached in the outpatient department at a tertiary eye hospital in London on several occasions, across different sub-specialties. Patients were taken to an empty clinic room, whilst waiting for their outpatient appointments, to complete the questionnaire.

### Data collection

Data collection took place between August and September 2020. The study followed the tenets of the Declaration of Helsinki, and all participants were provided with information relating to the questionnaire and the aims of the service evaluation. Participants who provided verbal consent were then proceeded to completing the survey. Participants were taken through the questionnaire by a researcher, who ensured that all parts of the questionnaire were completed. The participant responses were transcribed at the time of collection, and no recordings were obtained. We selected this method of data collection instead of distributing the questionnaire for participants to fill themselves as it ensures that all aspects of the questionnaire are answered and allowed us to collect more qualitative data. Participants were invited to review their responses after the completion of the survey. No identifiable data were collected. Each patient was allocated a unique survey ID number, written on their completed questionnaires. The researchers analysing the survey responses were not able to identify the patients from their survey ID numbers; hence, the questionnaire was anonymous to the analyst.

### Data analysis

Descriptive statistics of the study population were summarised. To investigate the factors correlating with attitudes to research, comparisons of demographics and baseline characteristics between patients who were willing and those not willing to participate in clinical trials were made using the chi-square tests (≤ 2 variables) and Fisher’s exact (> 2 variables) test for categorical characteristics and the Mann-Whitney *U* test for continuous characteristics. Statistical significance was defined as *p* < 0.05. The GraphPad Prism software version 9.0.0 was used for all statistical analyses. Excel version 16.63.1 was used to perform Cronbach’s alpha test to assess the internal consistency of the eight-item Likert-scale questions used in the survey and was also used to generate the figures.

Transcripts from each participant were analysed using an inductive thematic approach (as described by Kiger and Varpio) and facilitated by the Dedoose online software [19]. The initial codes were organised into emergent themes and sub-themes and then categorised independently by DA and HM and subsequently reviewed by TY to resolve any disagreements.

## Results

### Study population

A total of 53 patients participated in the survey. The median age of participants was 63 [47 to 70] years, and 51% were male. The median self-reported commuting time was 40 [20 to 50] min. The baseline characteristics are summarised in Table 1.

**Table 1:** Summary of patient descriptors

	Total	Willing to participate in trials (***n*** = 21)	Unwilling to participate in trials (***n*** = 32)	***P***-value
**Age** (years)	63.00	63.00	60.50	0.6039
**Commuting time** (min)	40.00	40.00	32.50	0.1561
**Gender**				
Male	27 (51%)	9 (43%)	18 (56%)	0.4064
Female	26 (49%)	12 (57%)	14 (44%)	
**Travels independently**	46 (87%)	18 (86%)	28 (88%)	> 0.999
**Level of education**				
Primary school	5 (9.4%)	3 (14%)	2 (6%)	0.6627
GCSEs	8 (15.1%)	2 (10%)	6 (19%)	
A/O levels	15 (28.3%)	6 (29%)	9 (28%)	
University degree	25 (47.2%)	10 (48%)	15 (47%)	
**Ethnicity**				
Caucasian	30 (56.6%)	13 (62%)	17 (53%)	0.7002
Asian	12 (22.6%)	5 (24%)	7 (22%)	
Afro-Caribbean	9 (17.0%)	2 (10%)	7 (22%)	
Others	2 (3.8%)	1 (5%)	1 (3%)	
**Employment status**				
Full-time	22 (41.5%)	10 (48%)	12 (38%)	0.3483
Part-time	2 (3.8%)	2 (10%)	0 (0%)	
Unemployed—seeking work	5 (9.4%)	1 (5%)	4 (13%)	
Unemployed—for health reasons	3 (5.7%)	1 (5%)	2 (6%)	
Retired	19 (35.8%)	7 (33%)	12 (38%)	
Student	2 (3.8%)	0 (0%)	2 (6%)	
**Current treatments**				
Eye drops	19 (35.8%)	7 (33%)	12 (38%)	0.6511
Laser therapy	8 (15.1%)	4 (19%)	3 (9%)	
Surgery in the last 6 months	14 (26.4%)	4 (19%)	10 (31%)	
None	27 (50.9%)	10 (48%)	17 (53%)	
**Number of co-morbidities**				
0	22 (41.5%)	8 (36%)	14 (64%)	0.3152
1	24 (45.3%)	13 (54%)	14 (46%)	
2	2 (3.8%)	0 (0%)	2 (100%)	
3	2 (3.8%)	0 (0%)	2 (100%)	
**Previous trial participation**				
Yes	9 (17.0%)	5 (56%)	4 (44%)	0.4637
No	44 (83%)	17 (39%)	27 (61%)	

### Willingness to participate in research

Participants were asked if they would be willing to participate in a clinical trial in the near future, and 40% indicated they would be willing; the remainder were unsure (28%) or completely unwilling (32%). The differences in the characteristics between those who were willing and those who were unwilling (including those who were unsure) are summarised in Table 1. There was no statistical difference across all baseline characteristics amongst the patients in the two groups.

### Understanding of involvement in clinical trials

41.5% of participants indicated that they did not know very much about clinical trials and could not offer an explanation. There was no significant difference between education level and the ability to offer an understanding of clinical trials (*p* = 0.7531). The views from the remaining participants were categorised into factors relating to the purpose of research and those relating to the process of research (Table 2).

**Table 2:** Participant understanding of involvement in clinical trials

Category	Theme	Codes	Example excerpt(s)
The purpose of clinical research	Resolution	Developing new treatments Addressing different questions Evidence-based medicine	P22: “a lot, novel therapy, all the clinical governing things like providing evidence-based medicine from a patient perspective it may help therapy and better treatment” P38: “taking a sample of patients for a particular reason”
	Innovation	Valuable part of science Improving management of current conditions Part of drug development	P18: “well, they try out different procedures and drugs on volunteered people, sometimes give a placebo and they won’t know which one” P20: “it is something we all have to be tested for to see if suitable to put on market”
	Progression	Improving patient care Improving services Helps the wider community	P1: “it’s necessary, have to have it to come up with treatments” P46: “testing out new treatments and advancing practice”
The process of clinical research	Participants	Experimentation Requires volunteers Animal testing	P9: “being guinea pigs” P22: “it depends on the trial; the first batch are people are those who put their necks on the line then after this it is safer” P34: “tested on animals and then on healthy people then on people whom the drug is demanded for”
	Intervention	Comparing two drugs Comparing two technologies	P48: “testing of new medication or technologies”
	Follow-up	Time-consuming process Rigorous process Need to stay in hospital for the duration of the trial	P4: “from my knowledge, people stay in hospital for trials to be done until it is released to wider community” P52: “done one before; it was too demanding for me”

*P[ ]* participant number

### Attitudes towards participating in clinical research

**Fig. 1:**
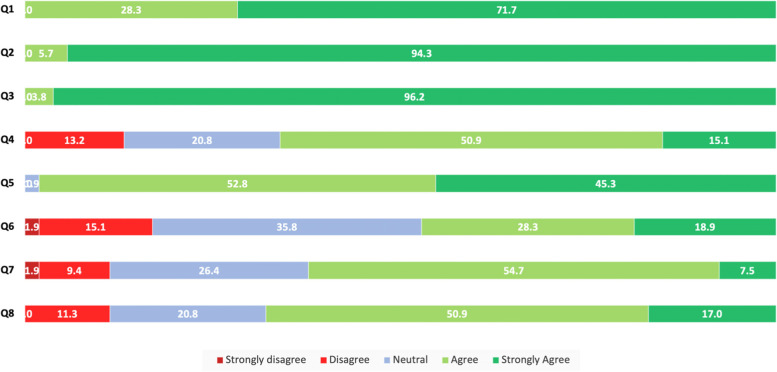
Participant responses to statements using a 5-point Likert scale, expressed as the percentage of the total number of participants (*n* = 53). *Q1: I think research into eye conditions is important*. *Q2: My vision is important to me*. *Q3: My eye care is important to me*. *Q4: I want to contribute to research*. *Q5: I believe results from trials could benefit patients in the future*. *Q6: I think the quality of care I receive will be better if I took part in a trial*. *Q7: If it’s possible to participate in research outside of hospitals, I would take part*. *Q8: I am more willing to participate in research if a mobile research unit was used*

The eight-part Likert scale questions yielded a Cronbach’s alpha score of 0.73, which is in the acceptable range. All patients agreed that research into eye conditions is important, and 98% agreed that the results from trials could benefit patients in the future (Fig. 1). However, only 66% wanted to contribute to the research. Less than half of the participants (47%) agreed that participating in trials would mean improved quality of care, and 79% wanted to minimise the amount of time spent in hospitals.

Participants were asked to suggest reasons they may want to participate in clinical research and which factors may prevent them from participating. Responses from the participants were categorised into motivating factors and those which prevented trial participation (Table 3). We identified four themes for motivating factors including personal gain, contribution to innovation, altruism and personal circumstances. Factors that prevented trial participation were divided into the following themes: fears around safety, inconvenience, lack of benefit, accessibility and personal experiences.

**Table 3:** Participant attitudes towards involvement in clinical trials

Category	Theme (***n***)	Sub-theme (***n***)	Codes	Example excerpt(s)
Motivating factors	Personal gain (16)		Aids own understanding of condition Find better treatments for own condition Financial reward Free services	P7: “help others, increase my own understanding of the eyes” P14: it will help people, it may improve my own treatment P18: “I think it could help my condition. Maybe reverse it and I am doing my best anything to enhance would be great” P51: “help keep my sight and improve it” P53: “for money and help research” P21: “depends on trial and if it’s in my interest improvement and well-being in life, if they can provide free services as incentives”
	Altruism (10)		Benefit to others Benefit to future generations	P7: “help others, increase my own understanding of the eyes” P13: “if there is, anyway, I can be helpful and anyone with my condition can help” P32: “help with future cure/treatment, research and interest” p48: “help future generations by coming up with new treatments”
	Contribution to innovation (12)		Improvement in management of conditions Help progression of field New solutions Sense of importance	P5: “it may help the development of medicine” P15: “it’s like learning, advancing science and better healthcare for people and for systems and can help people in hospital, helps remove excess burden” P30: “help improve medications” P31: “to help fellow people and advance medicine and science” P34: “help with the development of new drugs or treatment”
	Personal circumstances (2)		Lack of other options Benefit to relatives	P46: “no reasons at my age, worth trying anything” p48: “…last chance for my own health” P29: “can be helpful for my son”
Factors preventing participation in trials	Lack of benefit (4)		Lack of relevance to own condition Potential of no significant findings Inadequate financial incentives	P3: “depends on the topic, if I thought it was beneficial to me” P32: “no guarantee of treatment working” P52: “not enough money and possible infections”
	Inconvenience (27)		Time commitment Need to stick to appointments Interference with other activities Personal circumstances Commuting	P1: “wouldn’t have time, I do some part time work” P2: “don’t have time, I can’t be bothered and it’s too much hassle” P5: “time and personal circumstances, I have more operations coming up as does my wife” P13: “transport and getting there”
	Accessibility	Health condition (1)	Multiple co-morbidities	P27: “I am sick, I cannot move my arm after my stroke, my condition has already stopped me working” P10: “public transport is main concern”
		Current circumstances (4)	Difficulty in commuting	
	Personal experiences (10)		Multiple co-morbidities Bad experiences with medications Severity of condition	P19: “I have too many sicknesses” P43: “I’ve had a lot done to my eyes” P39: “bad experience with eye drops last year which was worrying” P49: “scared it might make my condition worse”
	Safety (31)	Current circumstances (8)	Fear of inadequate precautionary measures Minimise exposure to hospital environment	P14: “safety measures may not be taken” P21: “reluctance to take unnecessary risks especially in COVID and lockdown and going to places unnecessarily” P23: “I wouldn’t want to come into hospital more than I have to” P24: “possible side effects are worrying” P27: “I read about clinical trials at … Hospital, I do not want to be injected” P34: “worried about the life risk” P40: “depends on the stage of trials, not participate early on”
		Interventions used in the trial (23)	Fear of harmful effects Previous trials gone wrong Reluctance to join trial at an early stage	

*P[ ]* participant number

### Influence of the COVID-19 pandemic on attitudes towards participating in clinical research participation

Some of the factors preventing participation in trials were related to current circumstances and the COVID-19 pandemic. Participants expressed fear that trials may have inadequate precautionary measures and expressed the need to minimise exposure to the hospital environment in order to minimise risk (Table 3).

P14: “safety measures may not be taken”

P21: “reluctance to take unnecessary risks especially in COVID and lockdown and going to places unnecessarily”

P23: “I wouldn’t want to come into hospital more than I have to”

Some also expressed the impact that the pandemic may have on accessibility to hospitals for the purpose of research appointments, in particular, the need to avoid public transport and minimising exposure.

P10: “public transport is main concern”

Over a fifth (22.6%) of patients indicated that the COVID-19 pandemic has changed their outlook on research. Participant responses were categorised on positive and negative influences that the COVID-19 pandemic had (Table 4).

**Table 4:** Influence of the COVID-19 pandemic on participant attitudes towards research participation

Category	Theme	Codes	Example excerpt(s)
Positive influence	Increased awareness	Need for more research Realisation of the importance of research	P3: “could be more beneficial now” P4: “heightened my views” P37: “it will be more useful now”
	Altruism	Potential benefit to others	P26: “if I suffered from the virus then I may have wanted to help find a vaccine and go through the experience”
Negative influence	Safety	Avoiding unnecessary risks Fear of hospital environment	P21: “reluctance to take unnecessary risks especially in COVID and lockdown and going to places unnecessarily” P23: “more worried about the pandemic, I’ve been asked to shield”
	Emotional effects	Increased anxiety	P29: “made me more worried” P30: “worries me, don’t want to come into hospital”

*P[ ]* participant number

### Patient attitudes to attending hospital for clinical vs research appointments

Participants were asked which setting they would prefer to attend research appointments. Nineteen per cent have no preference, 32% wanted to attend them at a GP practice, 24% would rather attend them in a separate dedicated research unit and 25% would rather attend them in a hospital.

Attitudes to attending hospital for clinical appointments were overall positive, and the main reasons were the importance that face-to-face appointments had on the management of their condition and the confidence in the hospital to take the necessary precautions to ensure the safety of patients.

P8: “pleased to come as feel I need it badly”

P46: “have no problem at all, hospital takes good precautions”

Others expressed increased anxiety in particular with reference to the new format of delivering care and the potential of passing the virus on to family members.

P22: “anxious as I am unfamiliar with how the new clinic is being ran; it’s a new way of providing care”

P24: “very worried, this is my first time out of the house, I don’t leave without reason, my husband is disabled, and he may get it from me”

P36: “nervous, the risk and reward is not balanced for me”

Participant attitudes to attending hospital for research appointments as opposed to clinic appointments were mostly unaffected, but 41.5% indicated that they would not want to attend hospital for research appointments. Some of these reasons were expressed previously in their overall attitudes to clinical research. These included difficulty in commuting and accessibility, inconvenience and effort of participating in a trial, and potential for risks and, therefore, the need to minimise time spent in a hospital environment.

P26: “I am just here for hospital appointments”

P32: “I’d be worried in the current climate”

P23: “I wouldn’t want to come into hospital more than I have to”

P3: “I wouldn’t want to come in as it would take up too much time and effort”

## Discussion

This study aimed to investigate the factors that may influence a patient’s decision to participate in clinical trials and, in particular, whether the COVID-19 pandemic has had any influence on this decision. We found that 40% of patients were willing to participate in a hypothetical clinical trial, and this rate is lower than previous similar studies [10, 20, 21]. Similar to previous studies, all patients at least agreed that research is important and that results yielded from trials could be of benefit [10]. Yet, this proportion was not equally reflected in the numbers willing to contribute to research. This may be from previous negative experiences, as noted in our results. Similar to other studies, we found that motivating factors were driven principally by personal gain and contribution to innovation as well as altruism and personal circumstances [22]. Conversely, inhibiting factors were themed mainly around fears due to safety (both the environment and potential side effects from trial involvement) and inconvenience in addition to difficult accessibility and the potential for a lack of personal benefit. Bevan and colleagues had similar findings, with the most important factors to participating in research being altruistic motivations and personal benefits in helping their own treatment whereas the commonest deterrent factors were being too ill and not willing to change treatment and fear of side effects [20]. Few studies have been done to evaluate the factors motivating patients to enrol into clinical trials in the ophthalmology setting. Au and colleagues demonstrated that contribution to medical sciences and closer monitoring of conditions were the most commonly reported incentives for joining a trial [23]. It could be hypothesised from our study that although patients agree that research is important, the reason behind the lack of contribution is apprehension surrounding the current pandemic. Many felt as though attending hospital for clinical trials was not essential at this time and would ultimately put them at greater risk.

Conversely to certain studies, we found no association between demographic and disease factors and willingness to participate [10, 20]. Moreover, the level of educational background did not have an influence on the understanding of clinical trials. The root of patients’ perceptions towards clinical trials will be from their understanding of trials. We highlight some misconceptions in patients’ understanding of clinical trials, which may explain negative attitudes towards trial participation, namely the feeling of being experimented on and being treated as subjects rather than people. Prior studies have also demonstrated patient concerns regarding ethical concerns with RCT and highlight the importance of informed consent [23, 24]. Moreover, it has also been shown that patients often lack an understanding of their roles within a trial, and this may lead to reluctance in participation or a higher dropout rate [7]. This is further demonstrated in our study where previous trial participation had no significant impact on willingness to participate in a trial presently. Hence, the role of a patient-centred approach to trial conduct will be integral to improving attitudes. Patients are often aware of the benefits of trials, but the few anxieties they have may cloud their judgement. Our job as researchers is to iron out these fears and make the patient feel in control.

The COVID-19 pandemic has considerably disrupted clinical trial research, and this is particularly relevant given that trials often focus on patients who are most at risk from exposure to COVID-19 [8]. This is true for ophthalmology where a majority of trials are carried out on an older demographic given the risks of ophthalmic conditions (in particular, non-communicable eye diseases) increases with age. This is reflected in our study where the median age of patients is 63 years. This is the first study to demonstrate that where the COVID-19 pandemic had an effect on perceptions towards trial involvement, there was both a positive and negative influence noted. Interestingly, the setting of the study may be attributed to be the main factor which can be targeted by researchers to overcome the difficulties in patient recruitment as it affects both accessibility and the perceived safety risk to the patient (i.e. in minimising exposure to the hospital environment). Apprehension amongst patients is high when entering clinical environments, and it is the responsibility of the healthcare provider to ensure patients feel comfortable through the provision of personal protective equipment (PPE) and reassurance. The growing role of telecommunications and technology in healthcare provision may be one way to overcome this, for example, patients can be monitored during the course of a trial using remote technologies. However, this may be difficult to achieve in certain specialties where outcomes require face-to-face contact to be evaluated, especially in ophthalmology. Furthermore, patients who are elderly or sight-impaired may have difficulty using such technologies. Conversely, the pandemic has had a positive influence on some patients’ attitudes towards research, mainly by increasing the awareness of the importance of research in medicine and reinforcing altruism.

### Strengths and limitations

To our knowledge, this is the first prospective study investigating the factors that influence ophthalmic patients’ attitudes towards trial participation, and the first to assess these attitudes in the context of the COVID-19 pandemic. However, this study comprised a small sample size from a tertiary centre in ophthalmology. These results may not be extrapolated to patients with other conditions. This may, however, be offset by the richness of the qualitative data obtained from the survey by using open free-text questions. When completing the questionnaires, the researchers only read the questions verbatim outlined in the survey with no additional statements or clarifications. This is to reduce any potential influence the researcher may have on the responses attained from the participants. Another limitation in our study is the lack of patient involvement in designing the questionnaire which could have supported the relevance of our questionnaire to patient experiences further. Nonetheless, the use of open free-text responses increases the likelihood of capturing patient responses that would have otherwise been missed using multiple choice or scale-based questions.

## Conclusion

Understanding patient attitudes towards involvement in clinical trials is integral in trial design, which will in turn have invaluable effects on the recruitment and retainment of patients in clinical trials. Our findings demonstrate some misconceptions regarding the understanding of clinical trials, and this is likely the root of patient attitudes towards involvement in clinical trials. Adopting a patient-centred approach to the conduct of research will facilitate open communication and enhance understanding of involvement in trials. We must ensure the prompt return of non-COVID-19 trials, in particular, to those who partake in trials as the last resort with end-stage diseases [8]. The pandemic has had some positive influences on patient attitudes towards trials, but at the same time, it has highlighted certain barriers to trial conduct which must be addressed to enhance rates of trial participation, in particular, issues pertaining to accessibility and risks associated with being in a hospital environment.

## Supplementary Information

**Additional file 1.** Survey Questionnaire.

## Data Availability

The datasets during and/or analysed during the current study are available from the corresponding author upon reasonable request.
