# Supplementary material for: Factors impacting participation in research during the COVID-19 pandemic: results from a survey of patients in the ophthalmology outpatient department
Source: Trials. 2022 Sep 30;23:823. doi: 10.1186/s13063-022-06748-1 (PMC9522458; doi:10.1186/s13063-022-06748-1)
Supplement: Supplementary file 1 — Additional file 1. Survey Questionnaire. [file 13063_2022_6748_MOESM1_ESM.docx]

# **Participation in Ophthalmology Research Survey**

*Thank you for helping with our survey by telling us about your thoughts and experience of health research. We* *would like to understand what your thoughts are on research involvement. The* *information you give will be used to help improve the experience of people like you in future.*

*Completing this survey is entirely voluntary and we will not be asking for any personal details that will identify you. Your responses to the questions are in confidence and will not affect your treatment or prevent you from taking part in health research in future. The information we gather from you and others will not be used for any other purpose than to improve patient experience of* *health research in the NHS.*

# **Background questions**

| How old are you? | ______________ Years |
| --- | --- |
| What is your gender? | Male  Female  Other |
| What is your highest level of education? | Primary school  GCSEs  A/O- Levels  University degree |
| What is your ethnic group? | Caucasian  Afro-Caribbean  Asian  Other  Please specify if other: ________________ |
| What is your employment status? | Full time employment  Part-time employment  Unemployed – for health reasons  Unemployed – and seeking work  Retired  Student |
| Why are you here today? |  |
| How did you travel here today? | Public transport  Taxi  Bicycle  Foot  Car- driven self  Car- driven by friend/relative |
| Did you require any support to arrive here today? | Yes  No  If yes, please give further details: _____________________________________ |
| How long did your commute take? | Distance (approx.): _______________ Miles  Time (approx.): _____________Minutes |
| Which eye condition(s) are you diagnosed with? |  |
| Are you currently on any treatments for you eye condition(s), such as eye drops, lasers or operations? | Yes  No  If yes, please give further details (exact treatment and duration): _____________________________________  _____________________________________ |
| Which of the following medical conditions are you also being treated for? | No other medical conditions  High Blood Pressure  High Cholesterol  Diabetes  Mental health condition  Other  If other, please specify:  _____________________________________  _____________________________________ |

# **Involvement in clinical trials**

| What do you understand about being involved in a clinical trial? | |
| --- | --- |
|  | |
| Have you ever been offered to participate in a clinical trial before? | Yes  No  Don’t remember |
| Have you ever participated in a clinical trial before? | Yes  No  Don’t remember |
| If yes, how many trials? |  |
| If you have refused to participate in previous trials, please comment below you reasons why. | |
|  | |
| Would you be interested in participating in a clinical trial soon? | Yes  No  I’m not sure |
| What are some reasons you would want to participate?  Please write your answers in order of most to least important. | |
|  | |
| What are some reasons why you would not want to participate?  Please write your answers in order of most to least important. | |
|  | |
| Have your views on taking part in trials changed since the COVID-19 pandemic? | Yes  No  If yes, please give further details in the box below. |
|  | |
| How do you feel about coming into hospital during the COVID-19 recovery period?   1. For hospital appointments (clinic + surgery) 2. For research appointments | |
|  | |
| If you were to participate in a trial, where would you want to go for research appointments? | Hospital  GP practice  Separate dedicated research unit |

# **Perception of clinical trials**

Please indicate how strongly you agree or disagree with each of the following statements. **Please tick one box per statement.**

| **Statement** | **Strongly Agree** | **Agree** | **Neutral** | **Disagree** | **Strongly Disagree** |
| --- | --- | --- | --- | --- | --- |
| I think that research into eye conditions is important |  |  |  |  |  |
| My vision is important to me |  |  |  |  |  |
| My eye care is important to me |  |  |  |  |  |
| I want to contribute to research |  |  |  |  |  |
| I believe results from trials could benefit patients in the future |  |  |  |  |  |
| I think the quality of care I receive will be better if I took part in a trial |  |  |  |  |  |
| If it’s possible to participate in research outside of hospitals, I would take part |  |  |  |  |  |
| I am more willing to participate in research if a mobile research unit was used |  |  |  |  |  |

Any other comments?
